# Silicon Photonic Beam Steerer Based on Metalens Focal Plane Array

**DOI:** 10.1002/nap2.70274

**Published:** 2026-09-09

**Authors:** Chung‐Yu Hsu, Ping‐Yen Hsieh, Hsun‐Sung Chiu, Li‐Jun Tung, Chieh‐Chih Yu, Ko‐Chi Chen, Yu‐Heng Hong, Hao‐Chung Kuo, Chi‐Wai Chow, You‐Chia Chang

**Affiliations:** ^1^ Department of Photonics College of Electrical and Computer Engineering National Yang Ming Chiao Tung University Hsinchu Taiwan; ^2^ Semiconductor Research Center Hon Hai Research Institute Taipei Taiwan

## Abstract

Focal plane arrays (FPAs) promise robust solid‐state beam steering for LiDAR and free‐space optical communications. However, the need for external collimation lenses hinders chip‐scale compactness. Discrete switching between FPA elements further introduces blind spots and limits the number of resolvable points, restricting applications that require continuous tracking. Here, we demonstrate a silicon photonic beam steerer based on a metalens FPA that monolithically integrates the collimation lens on‐chip. Thermo‐optic prisms enable continuous fine‐tuning, eliminating blind spots and doubling the number of resolvable points. Continuous steering over a 62° field of view is achieved while maintaining high beam quality, with an average sidelobe suppression ratio of 19 dB.

## Graphical abstract

A silicon photonic beam steerer based on a metalens focal plane array (FPA) monolithically integrates the collimation lens on‐chip while enabling blind‐spot‐free continuous beam steering. Thermo‐optic prisms provide continuous fine‐tuning and more than double the number of resolvable points. Continuous steering over a 62° field of view is achieved while maintaining an average sidelobe suppression ratio of 19 dB.

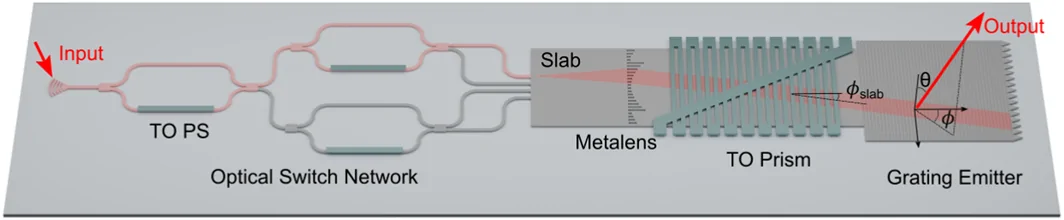

## 1 Introduction

Beam steerers are key components in scanning‐based technologies [1], such as light detection and ranging (LiDAR) [2, 3, 4, 5, 6], free‐space optical communications (FSO) [7, 8], and laser‐scanning microscopy [9, 10]. LiDAR enables 3D mapping of the physical world, holding great promise for autonomous vehicles, robotics, and the Internet of things. With the rapid advancement of artificial intelligence (AI), access to high‐quality 3D spatial information has become essential for training AI models and for enhancing the interaction between digital and physical domains. FSO can further establish highly flexible communications between spatially distributed nodes, including dynamic airborne platforms such as unmanned aerial vehicles (UAVs) [11]. These trends drive demand for ultracompact, highly reliable LiDAR and FSO systems. However, conventional mechanical beam steerers are too bulky to meet this demand, and their long‐term reliability is constrained by the moving parts that undergo repeated mechanical motion. Silicon photonics offers a solid‐state solution that miniaturizes a beam steerer to the chip scale with no moving parts. Thanks to its compatibility with the complementary metal‐oxide‐semiconductor (CMOS) processes, silicon photonics enables high‐yield integration of a large number of diverse components on the same chip. The ability to scale up the number of components in a circuit is particularly attractive as the resolution of beam steering depends critically on this number [12].

Optical phased arrays (OPAs) are among the most extensively explored beam‐steering technologies on the silicon photonic platform [3, 6, 7, 8, 9, 10, 13, 14, 15, 16, 17, 18, 19, 20]. In an OPA, light is distributed to multiple emitters, each with a controllable phase, to synthesize a steerable beam. Wide field‐of‐view (FOV) steering up to 180° [16, 17] and large‐scale integration with 9216 phase shifters have been demonstrated [18]. However, due to fabrication‐induced phase variations, phase calibration for a large number of phase shifters, typically performed using iterative algorithms or training, has been time‐consuming and unreliable [19]. For instance, calibrating a 128‐channel OPA can take ∼100 hours for data acquisition and training [19], and real‐time on‐chip phase monitoring remains challenging [20].

In contrast, focal plane arrays (FPAs) provide a more robust beam‐steering mechanism [2, 12, 21, 22, 23, 24, 25] as phase calibration is not required. An FPA comprises an optical switch network, an array of emitters, and a collimation lens. The optical switch network routes light to a selected emitter, whereas the lens—positioned at a focal length *f* from the emitter array—produces a collimated output beam. The beam direction is determined by the selected emitter location. Switch calibration is straightforward and relies solely on power monitoring. However, the discrete switching operation inherently limits steering to discrete angles, resulting in blind spots within the FOV. The discrete nature severely limits its applicability to FSO systems as link establishment requires fine alignment and tracking between transmitters and receivers. Moreover, the external collimation lens must be precisely aligned and packaged with the silicon photonic chip, adding system‐level bulk and increasing packaging cost [2, 5, 12, 21].

Another common limitation shared by both OPA and FPA technologies is that the number of resolvable points is approximately equal to the number of array elements *N* [12]. Consequently, achieving a practically useful high‐resolution beam steerer requires implementing a large‐scale array, which occupies significant chip area and complicates the control circuitry. This limitation can be mitigated by adopting irregularly spaced OPAs. For instance, employing a Costas array can enhance the number of resolvable points to *N* ^2^ [26]. However, this enhancement comes at the expense of beam quality and efficiency as sidelobe energy is redistributed across a broad angular range, resulting in elevated background noise.

Emerging on‐chip acousto‐optic beam steering offers a simple nonpixelated device architecture without requiring sophisticated control [4, 27]. It can generate multiple beams and independently modulate each beam through RF driving signals, making it particularly attractive for FSO. However, current implementations still require a bulky external lens for beam collimation, which limits the overall compactness of the system. Another emerging approach is microelectro‐mechanical systems (MEMS)‐based silicon photonic beam steering, which enables highly scalable steering with up to 128 × 128 resolvable angles and is therefore well suited for high‐resolution LiDAR applications [22]. However, because this approach is also based on an FPA architecture, it requires an external lens that must be precisely aligned and packaged with the silicon photonic chip. Furthermore, blind spots remain between adjacent pixels, which limits its suitability for FSO applications.

In this work, we present a solution to these long‐standing issues of FPA: we achieve continuous beam steering without blind spots and monolithically integrate the collimation lens on‐chip using metasurface technology [28, 29, 30]. A metalens FPA provides channel‐dependent discrete coarse steering, which is combined with a continuous fine‐tuning mechanism based on thermo‐optic (TO) prisms. This architecture increases the number of resolvable points by more than a factor of two without compromising beam quality. We experimentally demonstrate continuous steering over a FOV of 62°, while achieving an average sidelobe suppression ratio (SLSR) of 19 dB.

## 2 Operation Principle and Simulation

The schematic of the silicon photonic beam steerer is shown in Figure 1a. The device comprises an optical switch network, a metalens FPA, two TO prisms, and a shallow‐etched slab grating [28]. Azimuthal (ϕ) beam control is realized through discrete coarse steering by the metalens FPA and continuous fine‐tuning by the TO prism. In the altitude (θ) direction, wavelength‐dependent steering is achieved through dispersive diffraction from the slab grating. Optical microscope (OM) images of the device fabricated on a silicon‐on‐insulator (SOI) wafer are shown in Figure 1b–c. The 1 × 16 optical switch network routes the input light to a selected waveguide channel that terminates at a slab waveguide. The FPA is formed by 15 waveguide channels (3 μm wide, arranged at a 5 μm pitch) and a metalens. The metalens, positioned at a focal length of 200 μm from the terminations, collimates light launched from each channel. The on‐chip metalens, as shown by the scanning electron microscope (SEM) image in Figure 1d, is composed of subwavelength slots [31, 32, 33]. The metalens implements an optimized phase delay profile corresponding to an aberration‐free F‐theta lens [12]. When we launch light (in TE_0_ mode) from one of the waveguide channels, the metalens collimates it into a well‐defined propagation direction inside the slab. Channel selection determines the propagation direction (see the path in Figure 1a), enabling discrete coarse steering across 15 distinct angles. To enable continuous fine‐tuning of the beam direction, we implement microheaters—referred to as “thermo‐optic prisms”—to locally heat one of the two triangular regions of the silicon slab waveguide. Owing to the large thermo‐optic coefficient of silicon (${dn}_{\text{Si}}/dT$ = 1.86 × 10^−4^ K^−1^), localized heating increases the refractive index, forming a thermo‐activated prism that dynamically fine‐tunes the guided beam direction. Finally, the guided wave within the slab is diffracted to the air by the shallow‐etched slab grating, enabling beam emission with wavelength‐dependent steering.

**FIGURE 1:**
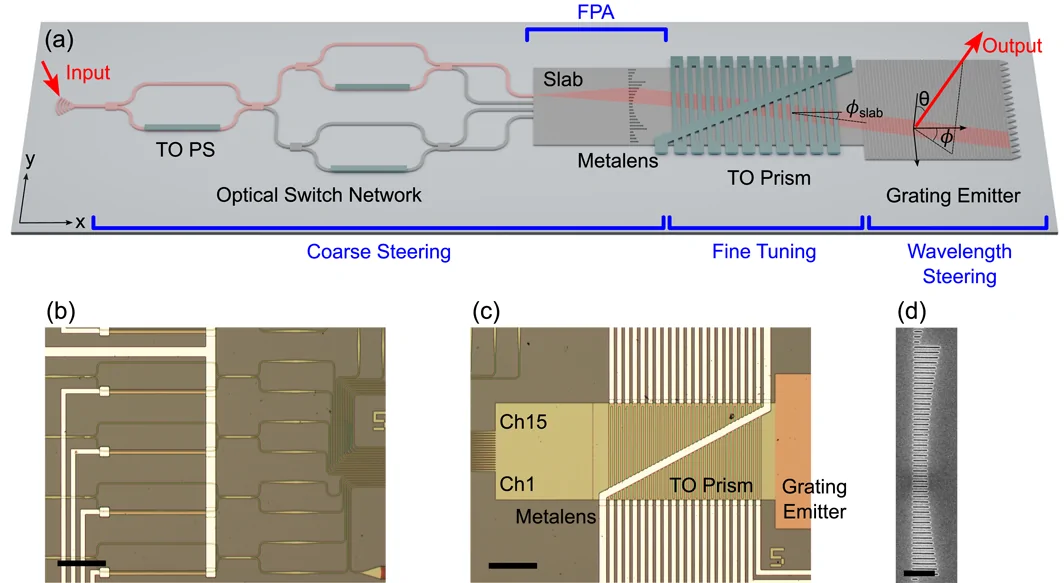
(a) Schematic of the silicon photonic beam steerer. FPA: focal plane array. TO: thermo‐optic. PS: phase shifter. Here, a simplified 1 × 4 optical switch network is plotted for clarity, whereas the actual device is 1 × 16. (b–c) Fabricated device under optical microscope, including (b) the optical switch network, and (c) metalens, TO prisms, grating emitter. Scale bar: 100 μm. Ch: channel. (d) SEM image of the on‐chip metalens. Scale bar: 3 μm.

We further elaborate on the beam‐steering mechanisms enabled by each component. To enable active switching, we incorporate an optical switch network for channel selection. As shown in Figure 1a, the optical switch network consists of Mach–Zehnder switches arranged in a binary tree structure. The Mach–Zehnder arms are balanced to ensure broadband operation and robustness against changes in the chip's base temperature. A 1 × N optical switch network has N−1 switches in total, whereas only $\log_{2}\;N$ switches need to be activated simultaneously to define the light path [12, 25]. In our demonstration, only 4 switches in the 1 × 16 optical switch network are activated at any given time, enabling low power consumption for coarse steering. We use 15 out of the 16 output channels for subsequent light routing. To reduce propagation loss, we use 1.2‐μm‐wide multimode waveguides to route most of the optical paths within the network. Single‐mode operation is maintained by employing adiabatic Euler bends [34].

We design an on‐chip metalens to achieve aberration‐free beam collimation, eliminating the need for packaging a bulky external lens. The metalens consists of subwavelength slots [31] with a period of 500 nm and a width of 140 nm. The slot length is varied in finite‐difference time‐domain (FDTD) simulations to construct a meta‐atom phase library, as shown in Figure 2a. By varying the slot length from 500 nm to 2.5 μm, the library covers a full 2π phase range, allowing the creation of any desired phase profile. The phase profile of an F‐theta lens is obtained through ray‐tracing optimization (see Supporting Information S1) [12], and the optimized profile is shown in Figure 2b. According to the ray‐tracing results, this design enables aberration‐free collimation up to a propagation angle of ± 9.9° within the silicon slab. We further validate the metalens FPA design using 3D full‐wave FDTD simulation. The far‐field angular distribution of the 15 channels is shown in Figure 2c, plotted as a function of $\phi_{\text{slab}}$, the propagation angle inside the silicon slab. The FDTD simulation confirms well‐collimated beams up to $\phi_{\text{slab}}$ = ± 9.9°, in agreement with the ray‐tracing design. Owing to the large refractive index contrast between silicon and air, the steerable angles in air after diffraction by the grating emitter are significantly larger than the ± 9.9° inside the slab, as discussed later. Figure 2d shows the simulated divergence angle and the beam‐forming efficiency (defined in Supporting Information S1). The average divergence angle inside the slab waveguide is 0.43°, corresponding to 1.22° in air. The beam‐forming efficiency reaches 79% at the central channel (Ch 8). Note that this efficiency reflects only the metalens' performance in forming collimated beams within the slab and does not include the grating emitter efficiency.

**FIGURE 2:**
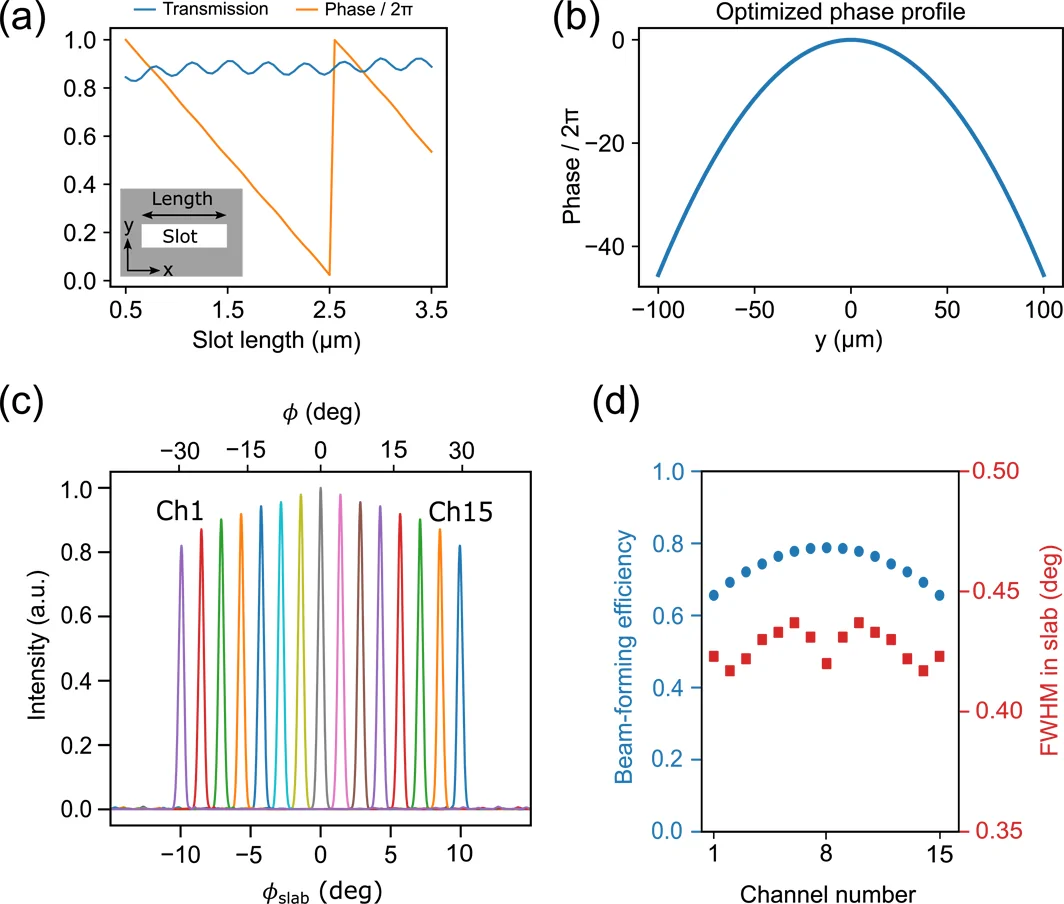
(a) Meta‐atom phase and transmission library. The inset shows the unit cell of the metalens. (b) Optimized phase profile of the F‐theta metalens. (c) FDTD‐simulated angular distribution of the wave in the slab waveguide after passing the metalens. Different colors represent launching from different channels. $\phi_{\text{slab}}$ (bottom horizontal axis) represents the azimuthal angle of the wave guided inside the silicon slab, while ϕ (top horizontal axis) represents the azimuthal angle for the emitted beam in air. (d) FWHM divergence angle inside the slab waveguide and beam‐forming efficiency of the metalens.

To enable continuous steering in the ϕ direction, we implement a fine‐tuning mechanism based on a pair of TO prisms formed by tungsten microheaters above the silicon slab waveguide as shown in Figure 3a. When current is applied, the microheaters locally heat a triangular region of silicon. Owing to the thermo‐optic effect, the heated region exhibits an increased refractive index and functions as a dynamic prism that deflects the guided beam. Similar concepts of thermo‐activated components have been demonstrated in other integrated photonic platforms [35]. The fine‐tuning mechanism introduced here closes the blind spots of the FPA‐based steering and increases the number of resolvable points. The same current is applied through 20 densely packed microheater sections (i.e., $I=I_{1}=I_{2}=I_{3}\text{⋯}$). The microheater sections are equally spaced and have the same width. Because the resistance of each section $\left(R_{i}\right)$ is proportional to its length ($l_{i}$), the dissipated heat per unit length (${I^{2}R}_{i}/l_{i}$) is constant for all sections. This design creates uniform heating across the triangular region (see Supporting Information S1 for detailed geometry). The two TO prisms, activated by the upper half and lower half of the microheater sections, respectively, share a common ground and can be independently activated. We then perform FDTD simulations to estimate the fine‐tuning angle as a function of the elevated temperature. To reduce computational cost, a smaller triangular region with the same slope is simulated (see Supporting Information S1). The simulated fine‐tuned propagation angle inside the slab waveguide as a function of the elevated temperature ΔT is shown in Figure 3b,c. The beam can be fine‐tuned in either the positive or negative direction, depending on which triangular region is activated. The angle change of the central beam exceeds its full width at half maximum (FWHM) when the elevated temperature is greater than approximately 60°C.

**FIGURE 3:**
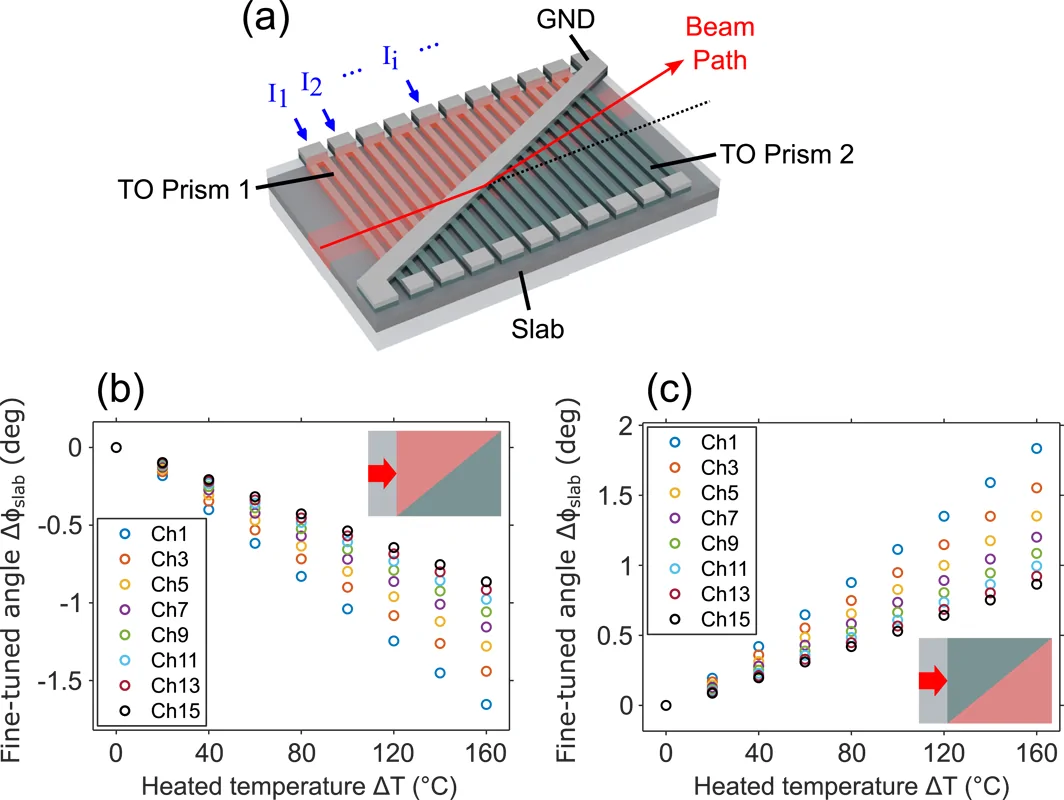
(a) Schematic of the TO prisms, which allow independent heating of two triangular regions of the slab waveguide to introduce fine beam deflection. The schematic is simplified for clarity, whereas in the actual device, each TO prism has 20 densely‐packed microheater sections. GND: ground. (b–c) Simulated fine‐tuning of the beam direction at different elevated temperatures with TO prism 1 (b) and TO prism 2 (c).

We employ a shallow‐etched grating emitter to diffract light from the guided slab mode into air and enable wavelength‐dependent steering in the altitude (θ) direction. The relationships between diffraction angles in air and the propagation angles inside the slab waveguide are given by

(1) $$k_{0}\;\sin\;\phi {=n}_{\text{slab}}k_{0}\;\sin\;\phi_{\text{slab}}$$

(2) $$k_{0}\;\sin\;\theta =n_{\text{slab}}k_{0}\;\cos\;\phi_{\text{slab}}-\frac{2\pi }{\Lambda }$$

Equation (1) represents Snell's law. Here, $n_{\text{slab}}$ and Λ denote the effective refractive index of the slab mode and the grating period, respectively. θ and ϕ are the altitude and azimuthal angles in air (defined in Figure 1a). Equation (1) is used to convert $\phi_{\text{slab}}$ to ϕ to predict the emission azimuthal angle in air as shown on the top horizontal axis of Figure 2c. In our architecture, $\phi_{\text{slab}}$ is actively controlled by the FPA and TO prism. Owing to the large refractive index contrast between silicon and air, ϕ is significantly larger than $\phi_{\text{slab}}$, enabling a large azimuthal FOV in air of up to ± 29.3° using coarse steering alone (see Figure 2c). The grating emitter has a period Λ = 580 nm and a duty cycle of 0.25, with a shallow etch depth of 20 nm to enable a large emission aperture. An antireflection subwavelength structure is incorporated at the end of the grating emitter as shown in Figure 1a. The emission θ angle at 1550 nm is designed to be 8.75°. The altitude FOV is determined by the grating dispersion and the laser tuning range.

## 3 Device Fabrication, Packaging, and Electrical Driving

We fabricate the beam steerer on a SOI wafer with a 220‐nm top silicon layer and a 3‐μm buried oxide (BOX) layer. Electron‐beam lithography (VOYAGER, Raith) is first used to define the fully etched waveguide layer with a negative photoresist (ma‐N 2403). The pattern is transferred into silicon via plasma etching (Plasma Pro 100 Estrelas, Oxford Instruments). A similar procedure is then performed using a positive photoresist (ZEP520A) to create the shallow‐etched grating. A 1.3‐μm‐thick silicon dioxide cladding is subsequently deposited by plasma‐enhanced chemical vapor deposition (PECVD) (Plasmalab 80 Plus, Oxford Instruments). On top of the cladding, we further sputter Ti/W with a total thickness of 143 nm and use lift‐off to create the microheater layer. Each TO phase shifter, formed by this Ti/W layer, has a width of 8 μm and a length of 200 μm. Finally, a 1‐μm‐thick aluminum layer is deposited by thermal evaporation and patterned via lift‐off to form the wires for electrical connection.

The fabricated chip is diced and mounted onto an aluminum holder using silver paste. We wire‐bond the chip to printed circuit boards (PCBs), which interface with a multichannel programmable voltage source (PXIe‐6739, National Instruments) for electrical driving as shown in Figure 4a. This voltage source provides 64 independent channels with an update rate of 350 kS/s, enabling control of 15 TO phase shifters in the optical switch network and 40 microheater sections in the TO prisms. For the TO prisms, we first measure the resistance of each section and then apply the corresponding driving voltage to generate a uniform temperature distribution across the triangular prism region.

**FIGURE 4:**
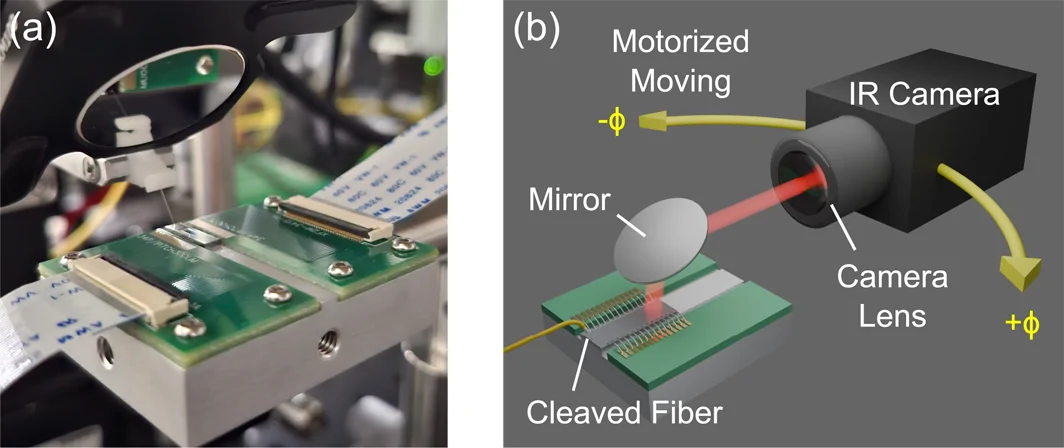
(a) Photography of the packaged chip. The silicon photonic chip is diced to a size of 10.5 mm × 3 mm. (b) Schematic of the setup for beam characterization.

## 4 Beam‐Steering Characterization

We experimentally demonstrate continuous beam steering over an azimuthal FOV of 62°. We characterize the beam‐steering performance by constructing a Fourier imaging system as illustrated in Figure 4b. A camera lens maps the 2D far‐field angular distribution onto an infrared camera (Bobcat 320, Xenics). We precisely rotate the system in the azimuthal direction using a motorized stage to allow measurements over a wide FOV. Light from a tunable laser (CTL 1550, Toptica) is coupled into the input grating coupler via a cleaved single‐mode fiber.

Figure 5a demonstrates the measured coarse steering in the ϕ direction and wavelength steering in the θ direction. The FPA‐based coarse steering generates 15 discrete beams controlled by the optical switch network. The wavelength is fixed at 1550 nm in this measurement. This figure also shows the dispersive beam steering in the θ direction as the wavelength is tuned to 1540, 1560, 1570, 1580, and 1590 nm. These wavelengths enable an altitude FOV of 8.3°, currently limited by the angular measurement range of the Fourier imaging system. The measured angular dispersion of the grating emitter is 0.17°/nm. In Figure 5b, we combine both FPA‐based coarse steering and TO‐prism‐based fine‐tuning to show continuous steering over an FOV of 62°. To characterize the fine‐tuning mechanism, we apply 1.3 W of electrical power to one of the TO prisms and measure the beam displacement for different channels as shown in Figure 5c. The fine‐tuning angle reaches 2.47° for Channel 1, corresponding to 152% of the beam's FWHM. For Channel 15, a smaller tuning angle of 1.35° is observed, consistent with the simulation shown in Figure 3. We further characterize the FWHM beam divergence angles and the fine‐tuning range of all 15 channels. As summarized in Section 12 of Supporting Information S1, 8 of the 15 channels exhibit a fine‐tuning range exceeding their FWHM divergence angle. Consequently, each of these channels supports three nonoverlapping beams: one at the original steering angle and two at the fine‐tuned steering angles in opposite directions. The remaining 7 channels achieve fine‐tuning ranges of at least 81% of their FWHMs. Based on the criterion of nonoverlapping FWHMs, the 8 channels produce 24 non‐overlapping beams, whereas the remaining 7 channels contribute 7 additional beams. In total, the proposed beam steerer achieves 31 resolvable points using 15 waveguide channels. Since conventional OPAs and FPAs are typically constrained to a number of resolvable points comparable to the channel count [12], this result represents more than a twofold enhancement in steering resolution. Based on the measured tuning efficiency, increasing the TO prism driving power to 1.6 W is expected to extend the fine‐tuning range beyond the FWHM beam divergence angle for all channels, thereby enabling 45 resolvable points and achieving threefold resolution enhancement. Figure 5d presents the fine‐tuning of the central beam (Ch 8) under different applied electrical powers to the TO prism, demonstrating continuous operation. Figure 5e presents the measured SLSR and FWHM divergence angles for different channels without fine‐tuning. The central beam (Ch 8) exhibits FWHM divergence angles of 1.45° in ϕ and 0.31° in θ. The measured average FWHM divergence angle in ϕ is 1.48°, slightly larger than the simulated value of 1.22°. The beam quality remains high as evidenced by the average SLSR of 19 dB.

**FIGURE 5:**
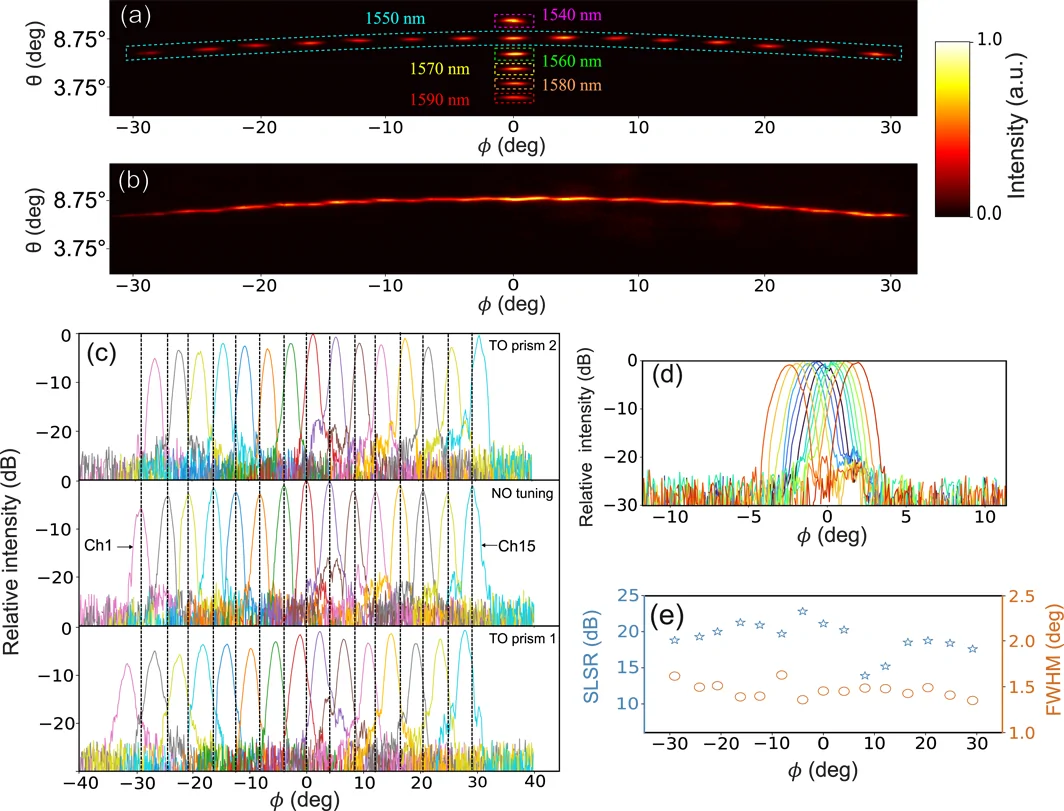
(a) Far‐field steering map demonstrating FPA‐based coarse beam steering with 15 channels and wavelength tuning. The map is produced by overlapping the images measured from 15 channels and 6 wavelengths. (b) Far‐field steering map combining FPA‐based coarse steering and TO‐prism‐based fine‐tuning, showing continuous steering over a 62° FOV. The map is produced by overlapping the images measured from 15 different channels and 3 different TO prism settings (1.3 W applied to TO prism 1, no fine‐tuning, and 1.3 W applied to TO prism 2). (c) Azimuthal cross‐section of the far‐field steering map showing both coarse steering and fine‐tuning. Different colors denote different channels. From top to bottom: 1.3 W applied to TO prism 2, no fine‐tuning, and 1.3 W applied to TO prism 1. (d) Fine‐tuning of the central beam under different applied electrical power to the TO prism. Power steps of 1.30, 0.92, 0.68, 0.48, 0.30, and 0.17 W are applied. (e) The measured SLSR and FWHM divergence angles for different channels.

We demonstrate programmable active beam steering and characterize the steering speed, achieving time constants on the order of 10 µs for both coarse steering and fine‐tuning mechanisms. By programming the multichannel voltage source and the tunable laser, the output beam can be steered along a predetermined trajectory. As an illustrative example, Figure 6a shows a steering map in which the beam traces the letter “H” in the far field. To characterize the steering speed, we aim the beam at a photodetector (PDA20CS2, Thorlabs) and modulate the beam direction using coarse steering and fine‐tuning mechanisms, respectively, while recording the photodetector response. The coarse steering speed is determined by the reconfiguration time of the optical switch network, governed by the TO phase shifters. As shown in Figure 6b, we reconfigure the optical switch network to move the beam into and out of the photodetector. The measured rise (heating) time, defined as the time from 10% to 90% of the signal amplitude, is 17.0 µs, whereas the fall (cooling) time is 8.8 µs. For fine‐tuning speed characterization, we first align the beam to the photodetector and then toggle the TO prism on and off. The result is shown in Figure 6c. The measured fall time (heating of the TO prism) is 15.1 µs, and the rise time (cooling) is 22.3 µs. Notably, despite the substantially larger heated area of the TO prism, its response speed remains comparable to that of conventional TO phase shifters.

**FIGURE 6:**
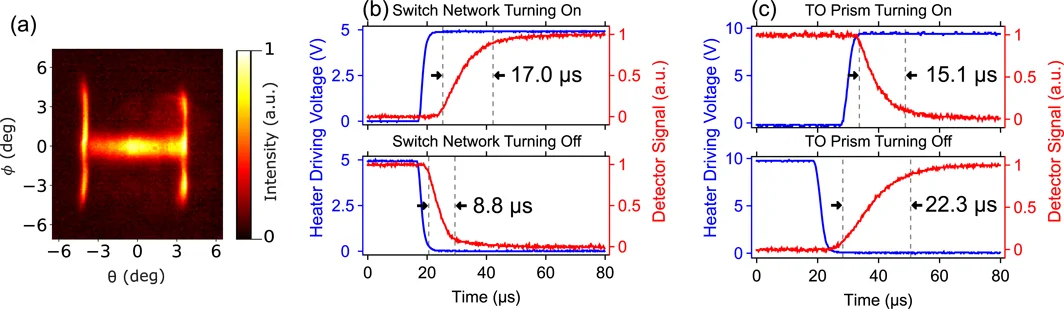
(a) Steering map that overlaps the scans with a trajectory tracing the letter “H”. (b) Coarse steering speed measurement. (c) Fine‐tuning speed measurement.

We further evaluate the power consumption and insertion loss of the silicon photonic beam steerer. The $P_{2\pi }$ of each TO phase shifter, defined as the electrical power required to induce a 2π phase shift, is 40 mW, a typical value for the silicon photonic platform [13]. The maximum power consumption of the optical switch network, consumed by $\log_{2}\;N$ switches, is 4 $\times P_{2\pi }$ = 160 mW. The measured extinction ratio of a single optical switch is 18.6 dB. The TO prism consumes up to 1.3 W of electrical power. As a result, the total electrical power consumption of the beam steerer, including both the optical switch network and the TO prism, has a maximum value of 1.46 W. The measured total insertion loss is 16.5 dB. A detailed breakdown of the insertion loss is provided in Supporting Information S1. The dominant contribution is the 10.5 dB insertion loss of the input grating coupler, which is primarily attributed to imperfections in our in‐house fabrication rather than the beam‐steering architecture itself. By employing a state‐of‐the‐art input grating coupler, the total insertion loss of the beam steerer could potentially be reduced to approximately 9 dB (see Supporting Information S1).

## 5 Discussions

We have demonstrated a silicon photonic beam steerer based on a novel FPA architecture. An on‐chip metalens eliminates the need for packaging a bulky external collimation lens. The incorporation of TO prisms removes blind spots, enables continuous fine‐tuning, and enhances the number of resolvable points by more than a factor of two. Continuous beam steering is particularly critical for FSO links, where fine alignment and tracking between transmitters and receivers are required [36]. High beam quality is maintained across the FOV as evidenced by an average SLSR of 19 dB. The architecture enables higher beam quality than conventional nonapodized OPAs, which have a theoretical maximum SLSR of 13 dB [16]. This architecture also offers more robust calibration compared to OPAs, as phase calibration is not required. The measured azimuthal FOV reaches 62°, currently limited by the aberration‐free numerical aperture of the metalens, which can be further increased using doublet designs [37] or optimization algorithms [38]. We demonstrate 31 resolvable points using only 15 waveguide channels, with the potential to increase this number to 45 through a modest increase in TO prism driving power. The architecture is readily scalable to a larger number of resolvable points by proportionally increasing both the channel count *N* and the metalens focal length *f* (see Sections 8 and 11 of Supporting Information S1). Currently, the altitude FOV is limited by the relatively low angular dispersion (0.17°/nm) of the shallow‐etched grating emitter. The angular dispersion can potentially be increased to ∼1°/nm using slow‐light photonic crystal waveguide gratings [39], or to 0.79°/nm using unidirectional grating emitters with large diffraction angles [40]. Overall, by combining external‐lens‐free integration, continuous steering, and high beam quality within a single silicon photonic platform, the proposed architecture opens a promising path toward practical beam steerers for next‐generation LiDAR and FSO systems.

## Author Contributions

**Chung‐Yu Hsu:** conceptualization, investigation, writing – original draft, methodology, validation, visualization, writing – review and editing, software, formal analysis, data curation. **Ping‐Yen Hsieh:** conceptualization, investigation, writing – original draft, methodology, validation, visualization, software, formal analysis, data curation. **Hsun‐Sung Chiu:** conceptualization, investigation, writing – original draft, methodology, validation, visualization, writing – review and editing, software, formal analysis, data curation. **Li‐Jun Tung:** methodology, validation, investigation. **Chieh‐Chih Yu:** methodology, investigation. **Ko‐Chi Chen:** methodology, investigation. **Yu‐Heng Hong:** resources, funding acquisition. **Hao‐Chung Kuo:** resources, funding acquisition. **Chi‐Wai Chow:** resources, funding acquisition. **You‐Chia Chang:** conceptualization, investigation, funding acquisition, writing – original draft, methodology, validation, visualization, writing – review and editing, software, formal analysis, project administration, data curation, supervision, resources.

## Funding

This work was supported by the National Science and Technology Council (113‐2622‐8‐A49‐013‐SB); National Science and Technology Council (111‐2221‐E‐A49‐014‐MY3); and National Science and Technology Council (114‐2221‐E‐A49‐039‐MY3).

## Disclosure

Y.C.C. and several coauthors are inventors on patent application(s) related to the beam‐steering technology described in this work. The intellectual property is assigned to National Yang Ming Chiao Tung University.

## Conflicts of Interest

The authors declare no conflicts of interest.

## Supporting information


Supporting Information S1

## Data Availability

Data underlying the results presented in this paper are not publicly available but may be obtained from the authors upon reasonable request.
