# Supplementary material for: Silicon Photonic Beam Steerer Based on Metalens Focal Plane Array
Source: Nanophotonics. 2026 Sep 9;15(17):e70274. doi: 10.1002/nap2.70274 (PMC13672134; doi:10.1002/nap2.70274)
Supplement: Supplementary file 1 [file NAP2-15-e70274-s001.docx]

Silicon Photonic Beam Steerer Based on Metalens Focal Plane Array: supplemental document

**1. Design of the phase profile of the F-theta metalens**

We use ray-tracing optimization to obtain the phase profile of an aberration-free F-theta metalens [1]. Prior to the ray-tracing design, we use 3D finite-difference time-domain (FDTD) simulation to calculate the mode expansion behavior from a 3 µm-wide waveguide to a large silicon slab waveguide. The equivalent numerical aperture (NA) is calculated to be 0.17 by fitting the divergent angle of the beam. Next, we use ray-tracing software and place point sources on the emission plane at 0 µm, 10 µm, 20 µm, and 30 µm from the optical axis to perform the optimization. As plotted in Fig. 2(b) of the main text, the optimized phase profile is given by

$\varphi\left( r \right)= -\text{7171}\left( r/R \right)^{2} + \text{2070}\left( r/R \right)^{4}-\text{1.09}\text{×10}^{\text{5}}\left( r/R \right)^{6}$

$+ \text{2.03}\text{×10}^{\text{6}}\left( r/R \right)^{8}-\text{8.53×}\text{10}^{\text{6}}\left( r/R \right)^{10}$,	(S1)

where $r$ is the distance from the center of the metalens, and $R$ is a normalized radius that equals 0.5 mm. This phase profile, according to the ray-tracing results, allows aberration-free beam collimation for the focal plane array (FPA) up to an output beam propagation angle of $\pm$9.9° in the silicon slab.

**2. Calculation of the beam-forming efficiency of the metalens**

We further verify the metalens FPA design by performing a two-stage 3D FDTD simulation. In the first stage, we simulate the expanding profile of the TE_0_ mode from a 3 µm-wide waveguide channel into the silicon slab and record the transverse electromagnetic fields after a propagation distance of 200 µm. In the second stage, the recorded fields are used as the source to the metalens, and far-field projection is performed after the light passes through the metalens. The far-field angular distribution from each channel is consistent with the ray-tracing design, as shown in Fig. 2(c) of the main text. To quantify the performance of the metalens, we define the beam-forming efficiency as follows:

$\text{Beam}\text{-}\text{forming efficiency}=\frac{\int_{\phi_{max}-3FWHM}^{\phi_{max}+3FWHM} I\left( \phi\right)d\phi}{P_{in}}$	. (S2)

$\phi_{max}$ is the angle at the peak of a specific channel. $FWHM$ is the full width at half maximum divergence angle of the channel. $I\left( \phi\right)$ is the intensity. $P_{in}$ is the input power to the metalens. The beam-forming efficiency indicates how much of the input light entering the metalens contributes to the collimated beam within $\pm3 FWHM$ range.

**3. Detailed microheater geometry of the thermo-optic prisms**

The microheater geometry of the thermo-optic (TO) prisms is shown in Fig. S1. It consists of 20 sub-heaters and a 15 µm-wide shared ground. Each sub-heater consists of two 4-µm-wide narrow heaters separated by 4 µm. The heated area is a triangle with a base of 331.4 µm and a height of 174.9 µm. The resistance is designed to be approximately 1 kΩ, allowing it to be driven by the multichannel programmable voltage source (PXIe-6739, National Instruments). Next, we place 20 identical sub-heaters in an array with a period of 16 µm, together with a shared ground along a diagonal from the lower left to the upper right, separating the structure into two triangles. This configuration forms two independently controllable TO prisms.

To operate the device, we apply the same current to each sub-heater to ensure uniform temperature across the entire region. At any given time, only one side is powered while the other side remains unpowered. As a result, a uniform temperature distribution is formed in the triangular region. The current is collected by the shared ground at the center.

Fig. S1. Illustration of the thermo-optic prisms in top view. The blue arrows indicate the current flow. We show only 20 channels in this illustration for simplicity, while the actual device has 40 channels. GND: ground.

**4. Simulation of the beam deflection by the thermo-optic prism**

The schematic of the simulation model is shown in Fig. S2, where the slab waveguide has a triangular region with a uniformly increased refractive index based on the thermal-optic coefficient of silicon. We use 2.5D FDTD to simulate a smaller triangular region (189 μm × 100 μm) with the same slope of 27.8$^{\circ}$ to save computational resources. Since the model is smaller than the actual TO prism, we launch a scaled Gaussian beam with a beam waist of 17.5 μm into a 100 μm × 220 nm silicon slab waveguide. The channel spacing is 2.5 μm in the y-axis. The far-field angle is calculated by capturing the electromagnetic fields after the heated triangular region.

Fig. S2. Simulation model of the thermo-optic prisms in the (a) longitudinal and (b) transverse cross-sections.

**5. Energy-efficient alternative design**

The high power consumption of the TO prism mainly arises from the need to heat a sufficiently large triangular region to enable beam deflection. This triangular region must fully cover the diameters of all steerable beams. Here, we present an example of a potential energy-efficient alternative that reduces the size of the TO prism and, consequently, its power consumption.

Figure S3(a) illustrates the original design with a metalens focal length of 200 μm. The diameter of the collimated beam at the output of the FPA is denoted by *d*. As an alternative, the design shown in Fig. S3(b) reduces the metalens focal length to 100 μm, thereby reducing the beam diameter to *d*/2. The TO prism maintains the same triangular shape (i.e., the same apex angles) to preserve the same beam-deflection performance, while its linear dimensions are reduced by a factor of 2. Consequently, the area of the TO prism is reduced by a factor of 4. However, the smaller beam diameter leads to a larger far-field divergence angle, which is undesirable. To compensate for this effect, a pair of metalenses—a negative metalens (Metalens 2) and a positive metalens (Metalens 3)—is introduced to form a 2X beam-expansion telescope. The telescope restores the beam diameter to *d* while reducing the beam-deflection angle by a factor of 2. As a result, the alternative design recovers the same beam performances as the original design, including the beam divergence angle and field of view (FOV).

Regarding the power consumption, two competing effects must be considered. The required power is reduced by a factor of 4 because of the smaller TO prism area, but increases by a factor of 2 because the telescope reduces the beam-deflection angles. Overall, the proposed design achieves a two-fold reduction in TO prism power consumption. The tradeoff is a higher optical insertion loss due to the additional pair of metalenses.

Fig. S3. (a) Schematic of the original design. (b) Schematic of the proposed energy-efficient alternative. Panels (a) and (b) are conceptual illustrations rather than rigorous device designs.

**6. Thermal crosstalk between channels**

Operating the TO prism for an extended period of time can increase the chip's base temperature. We have designed our optical switch network such that it is insensitive to changes in the base temperature. In the optical switch network, all the Mach–Zehnder switches are designed symmetrically with balanced arms. When the base temperature changes, both interferometer arms experience the same phase shift, resulting in no change in the output power. Therefore, the switching performance between channels is not affected by the operation of the TO prism. To experimentally verify this, we compare the far-field beam profiles of two adjacent channels, as shown in Fig. S4(a). We first switch the light to Ch 6 and operate the TO prism at 1.3 W for 3 minutes. We then switch the light to Ch 5 and turn off the TO prism. No thermal crosstalk is observed between these two adjacent channels. Furthermore, we label the beam positions corresponding to Ch 2–Ch 9 without fine-tuning. None of these beam positions exhibits observable thermal crosstalk.

Furthermore, the metalens performance is not affected by the increase in the chip's base temperature, according to finite-difference time-domain (FDTD) simulations. Figure S4(b) shows the simulated metalens libraries at two temperatures differing by 60 °C. The effect of temperature is incorporated through the thermo-optic coefficient of silicon. We observe only negligible changes in the phase and transmission libraries, indicating that the metalens performance is insensitive to temperature variations.

Fig. S4. (a) Measured far-field angular distributions. Red curve: Channel 5 without the TO prism activated. Blue curve: Channel 6 with 1.3 W applied to the TO prism. (b) Simulated metalens libraries at two different temperatures differing by 60 °C.

**7. Breakdown of the sources of insertion loss**

Here we provide a breakdown of the sources of insertion loss, as summarized in Table S1. We have fabricated a series of test keys in the same fabrication batch to extract the insertion losses of the major components. The insertion loss of the input grating coupler, extracted from a test key consisting of back-to-back grating couplers, is measured to be 10.5 dB. For the metalens, we design the test structure shown in Fig. S5, in which two identical metalenses form a 4*f* system. The average insertion loss of a single metalens, obtained by averaging the measurements over all channels, is 2.1 dB. Our beam steerer also includes a dedicated test input channel that bypasses the optical switch network and launches light directly into the slab waveguide of the metalens FPA. This test channel is used to measure the combined insertion loss of the metalens and the grating emitter, which is 5.5 dB. By subtracting the measured metalens loss, we estimate the insertion loss of the grating emitter to be 5.5 dB − 2.1 dB = 3.4 dB. The insertion loss of the optical switch network is estimated by subtracting the contributions of the input grating coupler, metalens, and grating emitter from the total insertion loss, yielding an estimated insertion loss of 0.5 dB.

We also characterize the waveguide propagation loss using a microring test structure. To reduce propagation loss, most of our circuit is implemented using multimode waveguides with a width of 1.2 μm, thereby reducing sidewall scattering loss [2]. These waveguides are connected using Euler bends to suppress the excitation of higher-order modes. The measured propagation loss of the multimode waveguide in the TE_0_ mode is ~1 dB/cm. It should be noted that the breakdown presented in Table S1 represents our best estimate based on measurements from different test structures. Due to fabrication nonuniformity in our in-house university cleanroom process, the values in Table S1 should be regarded as estimates rather than precise measurements.

The dominant contributor to the insertion loss is the input grating coupler. Owing to imperfections in our in-house fabrication process, the input grating couplers exhibit an insertion loss of 10.5 dB, which is significantly higher than the values typically reported in the literature. For example, imec reported a silicon-on-insulator (SOI) grating coupler with an insertion loss of 1.6 dB in Ref. [3]. Therefore, the high insertion loss is mainly attributed to the performance of our fabricated grating couplers rather than the beam-steering architecture itself. By employing grating couplers with state-of-the-art performance, the total insertion loss of the beam steerer could potentially be reduced to approximately 9 dB.

Fig. S5. The layout of the test structure for the metalens. Two metalenses are identical and form a 4*f* system. GC: grating coupler.

Table S1. The breakdown of the insertion loss.

| Components | Insertion loss |
| --- | --- |
| Input grating coupler | 10.5 dB |
| Optical switch network | 0.5 dB |
| Grating emitter | 3.4 dB |
| Metalens | 2.1 dB |
| Total loss | 16.5 dB |

**8. Scaling of the power consumption**

Here we estimate the power consumption of our architecture when scaled to achieve approximately one thousand resolvable points. If the same waveguide channel design (3 μm wide with a 5 μm pitch) is maintained and the number of channels is increased to 335, the number of resolvable points increases to 1005. Compared with the original 15-channel design, this corresponds to a scaling factor $S$ of 22.3. The focal length of the metalens scales by the same factor $S$, increasing from 200 μm to 4467 μm. The TO prisms maintain the same triangular shape (i.e., the same apex angles), while their linear dimensions scale with the scaling factor. The area of the TO prism scales with $S^{2}$. Consequently, the enlarged TO prisms require higher electrical power. On the other hand, the full width at half maximum (FWHM) divergence angle of the emitted beam decreases with the scaling factor $S$ because it is inversely proportional to the aperture size. The reduced divergence angle decreases the required fine-tuning range of the TO prism, thereby reducing the power required to achieve additional resolvable points.

Considering both effects—the increased TO prism area and the reduced divergence angle—the power consumption of the TO prism scales linearly with the scaling factor $S$. For the example of 1005 resolvable points, the maximum total power consumption of the beam steerer is estimated to be 36 W, including contributions from the TO prism and the optical switch network. This analysis is intended to illustrate the scaling trend of the current architecture. The energy-efficient alternative design is discussed in Section 5 of the Supplemental Information.

**9.** **Expansion of the aperture sizes for long-range applications**

We estimate the effective emitting aperture size of our device from the measured far-field divergence angle. Assuming a Gaussian beam, the relationship between the Gaussian beam diameter $d$ and the FWHM divergence angle $\theta_{\text{FWHM}}$ is given by

$d=2\sqrt{2ln2}\frac{\lambda}{\pi\theta_{\text{FWHM}}}$,	(S3)

where the factor of $\sqrt{2ln2}$ is used to convert between the FWHM and 1/e^2^ size definitions. Using this relationship, we convert the measured divergence angles into the equivalent Gaussian beam diameters (i.e., the aperture sizes). In the $\phi$ direction, the measured divergence angle is 1.48°, corresponding to an effective aperture size of 45 µm. In the $\theta$direction, the measured divergence angle is 0.31°, corresponding to an effective aperture size of 215 µm.

For long-range LiDAR and FSO applications, a larger emitting aperture is required to further reduce the beam divergence. Here, we use the results reported in Ref. [4] as a benchmark to estimate the required aperture size. Reference [4] experimentally demonstrated 22 m ranging with a beam divergence of 0.16° × 0.04°. To achieve a comparable beam divergence, the aperture of our device would need to be increased by a factor of 9.3 in the $\phi$ direction and 7.8 in the $\theta$ direction, resulting in an effective aperture size of 419 μm × 1677 μm. The aperture expansion in the $\phi$ direction can be achieved by proportionally increasing the focal length of the metalens to 1860 μm. In the $\theta$ direction, the larger aperture requires a weaker grating strength, which can be realized using SiN gratings [4] instead of the silicon gratings employed in our work, or by incorporating SiN overlays on silicon waveguides [5].

**10. On-chip monitoring for long-term robustness**

Based on our previous experience with similar Ti/W heaters fabricated by our group, we have observed a slow drift in the heater resistance over a period of several months. However, we believe that this slight resistance drift does not compromise the robustness of the proposed solid-state beam steerer.

In particular, the FPA-based architecture readily supports on-chip monitoring and calibration. As illustrated in Fig. S6, on-chip photodetectors can be incorporated to monitor the optical power in each channel, enabling calibration of the optical switch network by updating the driving signals. A similar monitoring scheme can also be implemented to calibrate the fine-tuning angle provided by the thermo-optic prism, as shown in Fig. S6. These calibration procedures can compensate for gradual resistance drift by adjusting the driving signals accordingly, thereby maintaining stable beam-steering performance over time.

Fig. S6. Schematic of the proposed device structure incorporating on-chip monitoring. GC: grating coupler. PD: photodetector.

**11. Scalability limits: chip area and phase error accumulation**

Here we discuss how the proposed architecture can be scaled to support practical high-resolution LiDAR systems requiring more than 1000 resolvable points. The dimensions of the FPA scale linearly with the number of resolvable points. If the same waveguide channel design (3 μm wide with a 5 μm pitch) is maintained and the number of channels is increased to 335, the number of resolvable points increases to 1005. Compared with the original 15-channel design, this corresponds to a scaling factor of 22.3. The focal length of the metalens scales by the same factor, increasing from 200 μm to 4467 μm. Consequently, the slab waveguide area between the waveguide channel terminations and the metalens increases from 200 μm × 200 μm to 4467 μm × 4467 μm.

Light propagating in the slab waveguide accumulates more phase error as the area increases in the presence of slab-thickness nonuniformity. This effect imposes a practical limit on the maximum size of an on-chip FPA architecture. At present, we have not performed a rigorous analysis to determine whether a slab area of 4467 μm × 4467 μm is feasible, as this is beyond the scope of the present work. Nevertheless, we can compare the required device area with other large passive devices implemented on the silicon-on-insulator (SOI) platform. For example, an arrayed waveguide grating (AWG) with a footprint as large as 2.3 mm × 2 mm has been demonstrated [6]. Moreover, unlike an AWG, in our architecture the phase error arises solely from silicon-thickness nonuniformity in the original SOI wafer, not from subsequent lithography and etching processes. Therefore, we expect phase-error accumulation in the proposed architecture to be less severe than that in an AWG, suggesting that an FPA with dimensions of 4467 μm × 4467 μm should be achievable.

**12. Experimental determination of the number of resolvable points**

Figure S7 shows the measured far-field angular distributions of all channels under fine-tuning with 1.3 W applied to the TO prism. For each channel, we characterize the FWHM beam divergence angle and the fine-tuning range. As summarized in Table S2, 8 of the 15 channels exhibit a fine-tuning range exceeding their FWHM beam divergence angle, thereby producing three non-overlapping beam positions. The remaining 7 channels achieve a fine-tuning range of at least 81% of their FWHMs. To make our claim rigorous, we count only beam positions with rigorously non-overlapping FWHMs. Consequently, the 8 channels produce 24 non-overlapping beam positions, while the remaining 7 channels contribute 7 additional beam positions. In total, we achieve 31 resolvable points using 15 waveguide channels, corresponding to a resolution enhancement of more than twofold.

We would also like to point out that the driving power of the TO prism can be straightforwardly increased from 1.3 W to 1.6 W. This would allow the fine-tuning range of every channel to exceed its FWHM beam divergence angle, thereby enabling 45 rigorously resolvable beam positions and achieving threefold enhancement. However, in the current experimental setup, the multichannel voltage source (PXIe-6739, National Instruments) has already reached its maximum output power.

Fig. S7. Measured far-field angular distributions of all channels under three TO prism settings used to evaluate the fine-tuning range. The blue, red, and green curves correspond to 1.3 W applied to TO prism 1, no fine-tuning, and 1.3 W applied to TO prism 2, respectively.

Table S2. Summary of the FWHM beam divergence angles and fine-tuning ranges of each channel.

| Channel | FWHM | Fine-tuning range (one side) | Fine-tuning range/FWHM |
| --- | --- | --- | --- |
| 1 | 1.62$^{\circ}$ | 2.47$^{\circ}$ | 152 % |
| 2 | 1.50$^{\circ}$ | 2.14$^{\circ}$ | 143 % |
| 3 | 1.51$^{\circ}$ | 1.85$^{\circ}$ | 122 % |
| 4 | 1.40$^{\circ}$ | 1.81$^{\circ}$ | 129 % |
| 5 | 1.62$^{\circ}$ | 1.61$^{\circ}$ | 99 % |
| 6 | 1.37$^{\circ}$ | 1.52$^{\circ}$ | 111 % |
| 7 | 1.45$^{\circ}$ | 1.33$^{\circ}$ | 91 % |
| 8 | 1.46$^{\circ}$ | 1.18$^{\circ}$ | 81 % |
| 9 | 1.48$^{\circ}$ | 1.52$^{\circ}$ | 102 % |
| 10 | 1.48$^{\circ}$ | 1.47$^{\circ}$ | 100 % |
| 11 | 1.43$^{\circ}$ | 1.29$^{\circ}$ | 90 % |
| 12 | 1.49$^{\circ}$ | 1.33$^{\circ}$ | 89 % |
| 13 | 1.42$^{\circ}$ | 1.24$^{\circ}$ | 87 % |
| 14 | 1.35$^{\circ}$ | 1.16$^{\circ}$ | 86 % |
| 15 | 1.26$^{\circ}$ | 1.35$^{\circ}$ | 107 % |

**References**

1.	Y. C. Chang, M. Chul Shin, C. T. Phare, S. A. Miller, E. Shim, and M. Lipson, "2D beam steerer based on metalens on silicon photonics," Opt. Express **29**(2), 854 (2021).

2.	A. Rizzo, U. Dave, A. Novick, et al., "Fabrication-robust silicon photonic devices in standard sub-micron silicon-on-insulator processes," Opt. Lett. **48**(2), 215-218 (2023).

3.	D. Vermeulen, S. Selvaraja, P. Verheyen, G. Lepage, W. Bogaerts, P. Absil, D. Van Thourhout, and G. Roelkens, "High-efficiency fiber-to-chip grating couplers realized using an advanced CMOS-compatible Silicon-On-Insulator platform," Opt. Express **18**, 18278 (2010).

4.	B. Chen, Y. Li, Q. Xie, et al. "SiN‐On‐SOI optical phased array LiDAR for ultra‐wide field of view and 4D sensing," Laser Photonics Rev. **18**, 2301360 (2024).

5.	M. Zadka, Y. C. Chang, A. Mohanty, C. Phare, S. P. Roberts, M. Lipson, "On-Chip Platform for Phased Array with Minimal Beam Divergence and Wide Field-of-View, " Opt. Express **26,** 2528 (2018).

6.	Y. Liu, X. Wang, Y. Yao, J. Du, Q. Song, and K. Xu, "Silicon photonic arrayed waveguide grating with 64 channels for the 2 µm spectral range," Opt. Lett. **47,** 1186-1189 (2022).
